# Optimizing Variational Graph Autoencoder for Community Detection with Dual Optimization

**DOI:** 10.3390/e22020197

**Published:** 2020-02-07

**Authors:** Jun Jin Choong, Xin Liu, Tsuyoshi Murata

**Affiliations:** 1 Department of Computer Science, Tokyo Institute of Technology, Tokyo 152-8552, Japan; murata@c.titech.ac.jp; 2 National Institute of Advanced Industrial Science and Technology, Tokyo 135-0064, Japan; xin.liu@aist.go.jp

**Keywords:** variational inference, graph neural network, variational autoencoder, network embedding

## Abstract

Variational Graph Autoencoder (VGAE) has recently gained traction for learning representations on graphs. Its inception has allowed models to achieve state-of-the-art performance for challenging tasks such as link prediction, rating prediction, and node clustering. However, a fundamental flaw exists in Variational Autoencoder (VAE)-based approaches. Specifically, merely minimizing the loss of VAE increases the deviation from its primary objective. Focusing on Variational Graph Autoencoder for Community Detection (VGAECD) we found that optimizing the loss using the stochastic gradient descent often leads to sub-optimal community structure especially when initialized poorly. We address this shortcoming by introducing a dual optimization procedure. This procedure aims to guide the optimization process and encourage learning of the primary objective. Additionally, we linearize the encoder to reduce the number of learning parameters. The outcome is a robust algorithm that outperforms its predecessor.

## 1. Introduction

Networks (graphs) with nodes (vertices) and edges (links) are a considerable simplification of complex patterns observed in real life, thus permitting studies of complex systems. For instance, the study of social interactions between individuals can be represented in the form of social networks [1]. Researchers who published together can be related in a collaboration network [2]. Movies and their respective critics can be presented as a bipartite graph with the edge-weight indicating a user-movie rating [3] which further allow applications like recommender systems [4]. The flexibility of networks and its vast literature on graph theory makes network science [5,6] extremely appealing to researchers.

An area of interest with significant importance is community detection, also known as graph clustering [7], i.e., identifying groups of densely connected nodes. Traditionally, researchers have measured communities in terms of partition quality, known as modularity [8]. A recovered community structure with high modularity implies good partitioning. To this date, community detection algorithms have evolved from traditional algorithms to the usage of complex learning algorithms like graph representation learning [9,10]. In graph representation learning, one can enforce nodes within the same community to share similar representations. These representations are learned by aggregating features from neighboring nodes. In addition, graph representation learning is extremely appealing because it provides a generalized application for downstream tasks such as link prediction [11], classification [12] and clustering [13]. By exploiting existing literature on representation learning, these tasks can be solved simply by reusing existing machine learning techniques.

Among many types of graph representation learning algorithms, Graph Neural Network (GNN) has recently gained significant popularity. Inspired by Deep Learning methodologies, GNN is designed to follow a similar learning approach, but with graphs as its primary application. For instance, in graphs, convolutional layers are replaced with graph convolutional layers [14]. The outcome is a translation of Deep Learning techniques from computer vision readily applied to graph data. Likewise, GNN inherits similar disadvantages from deep learning algorithms, which is widely known to be a black-box learning algorithm. To overcome this problem, machine learning researchers have explored explainable artificial intelligence (XAI) algorithms. Causal inference [15] and Bayesian Deep Learning [16,17] are some examples of attempts to unravel the mysteries behind machine learning algorithms by presenting uncertainties and causal reasons.

From a different paradigm, generative models are equally appealing for introducing explainability. Stochastic Blockmodel (SBM) [18,19] is a popular approach to model networks. By proposing an assortative configuration on the stochastic matrix, one can generate networks that exhibit community structures. Leveraging on the reparameterization trick, Variational Autoencoder (VAE) [20] improves explainability by introducing uncertainty to an autoencoder. Recently, Kipf and Welling [21] proposed Variational Graph Autoencoder (VGAE), which results in research variants such as VGAECD [22] and ARVGA [23].

**Figure 1:**
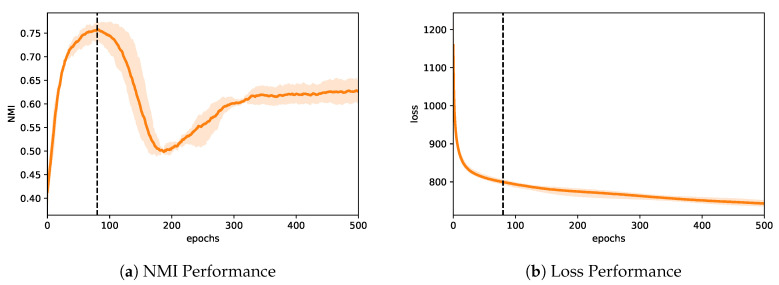
Left: The deviation problem exhibited when training VGAECD. The NMI drops approximately after 80 epochs and gradually begins its re-ascent. In most cases, it deteriorates in favor of its secondary objective of minimizing the reconstruction loss. Right: The performance of loss continues to drop regardless of its NMI performance.

Albeit powerful, VGAE-based algorithm suffers from an optimization problem. When trained, it has tendencies to deviate from its primary objective in favor of the reconstruction of the input network and eventually lead to a posterior collapse [24]. In this work, we focus our attention on a variant of VGAE, namely Variational Graph Autoencoder for Community Detection (VGAECD) [22]. We found that optimizing the loss using stochastic gradient descent often leads to sub-optimal community structure when the model is initialized poorly. Figure 1 demonstrates an example of the quality of detected communities (measured by NMI) with respect to the loss during training in a synthetic network generated by the LFR benchmark with μ=0.60. We can observe that the training loss is consistently decreasing as expected. However, the NMI suddenly drops approximately after 80 epochs and gradually begins its re-ascent. This tendency has also been observed in other unsupervised deep learning algorithms [25,26,27]. To circumvent this problem, one can train the unsupervised algorithm with a meta-learner [25,26]. More specifically, one can introduce a guide that prevents the algorithm from going astray. This new formulation comes with an advantage and disadvantage. Specifically, the weakness comes at the cost of more computation complexity.

Additionally, generalization becomes difficult because of the high coupling between the learner and the task of interest (task-dependent). Instead, in our work, we chose to leverage a variational solution. More specifically, we maximize the lower bound introduced in the Variational Autoencoder such that no additional modification is required on the original loss function. Instead, the optimization procedure follows a Neural Expectation-Maximization (NEM) algorithm [28], which guarantees that communities do not collapse. This is possible because NEM can be formulated in terms of maximizing a variational lower bound [29]. Maximizing this lower bound ensures that every new update is an improvement over the previous step. Furthermore, it has a theoretical guarantee for convergence up to local optima. We term our improved version, Variational Graph Autoencoder for Community Detection - Optimized (VGAECD-OPT).

To summarize, we improve VGAECD and propose a robust algorithm (VGAECD-OPT) for community detection. Our contributions are as follows,

- Demonstrate the efficacy of linearization on VGAECD’s encoder in community detection task [30,31].
- Propose a dual optimization approach to alleviate the deviation of objective functions (community detection vs. network reconstruction)

The proposed algorithm, like VGAECD, inherits properties unique to generative models such as the possibility to generate a synthetic network with community structure. Such models can be useful to application areas such as high-performance computing [32,33]. On the other hand, the model itself can be used as a network anonymizer by inducing *artificial* links or nodes on an existing social network [34].

## 2. Related Work

From a probabilistic modeling perspective, community detection can be divided into two classes, namely discriminative and generative models. The former is a class of algorithms that attempt to maximize the community structure recovery while the latter considers the process of generating a network that exhibits community structure with high fidelity. In this section, we briefly explain recent work on these algorithms.

### 2.1. Discriminative Models

Traditionally, community structures were identified via connectivity patterns such as density within a community [35,36]. In practice, these patterns can be measured by a quality metric such as modularity [8] and conductance [37]. The Louvain method [38], is a greedy algorithm that maximizes the modularity objective function. Although popular, modularity maximization is known to exhibit a resolution limit [39] and degeneracies [40]. On the other hand, propagation algorithms such as label propagation [41] are popular for detecting communities in networks at scale. Other approaches such as WalkTrap [42] and Infomap [43] balances scalability with computational performance [44]. Representation learning methods such as GraRep [45] and CFOND [46] cast community detection as a matrix factorization problem. Models like GA-Net [36] employ a traditional genetic algorithm while maximizing a *community score* defined on the maximization of the dense internal sub-matrices. Moscato et al. [47] formulates community detection as a game model, employing Game Theory approaches to maximize the community assignment.

Recent successes [48,49] in deep learning rekindled interest in unsupervised learning models such as autoencoders for networks. In particular, GraphEncoder proposed by Tian et al. [50] showed that optimizing the objective function of the autoencoder is similar to finding a solution for Spectral Clustering [50]. Leveraging on deep learning’s non-linearity and recent advances in Convolutional Neural Networks, Defferrard et al. [51] proposed Graph Neural Network (GNN), with Kipf and Welling [14] further simplifying to Graph Convolutional Neural Network (GCN).

On the other hand, DeepWalk [12] and node2vec [11] are popular algorithms for graph embedding. To generate a co-occurrence context, random walks are used in conjunction with negative sampling for large scale datasets. More recently, this line of algorithms can be generalized to a class of matrix factorization algorithms [52,53]. Albeit powerful, the model has many hyperparameters to tune, which can be time-consuming.

### 2.2. Generative Models

Generative models can be classified into two types: algorithmic and statistical models. In the former case, graphs are generated under assumptions of prior knowledge. For instance, Kronecker Graphs [54] considers the generation of graphs via a Kronecker product. The Block Two-Level Erdős-Rényi (BTER) model [55] considers a greedy approach for matching clustering coefficient and degree distribution. Simpler models include benchmark graph models such as Girvan-Newman [35], Lancichinetti–Fortunato–Radicchi (LFR) Graph [56] and mLFR [57]. The latter considers a class of algorithms from the lens of probabilistic graphical modeling. Given a network as the input, its goal is to maximize the likelihood of the latent variables which generate the same input network. For example, the Stochastic Blockmodel (SBM) [18] considers a stochastic matrix B as the probability of connectivity under the assumption of stochastic equivalence (i.e., nodes within the same community shares the same connectivity pattern). Karrer and Newman [19] further extends this work to community detection by introducing a degree correction procedure to the algorithm. Extensions to SBM includes the Mixed Membership SBM (MMSBM) [58] for identifying mixed community participation and bipartite SBM (biSBM) [59] for finding communities in bipartite networks. Today, SBM is well explored, and its limitations have been widely studied [60,61]. However, SBM is not a network representation learning model. In other words, SBM’s learning paradigm differs from representation learning, which is the goal of this work.

From the lens of representation learning, autoencoders are considered the closest cousin to generative models. With an encoder and decoder framework, it is no surprise that one considers autoencoders as a generative model. In reality, the autoencoder lacks sampling capability, which is the core of a generative model. To alleviate this problem, recent literature considers alternative models such as Generative Adversarial Networks (GAN) or Variational Autoencoder (VAE), which introduces an approximate posterior. For graphs, Kipf and Welling [21] introduced a variant of VAE for link prediction tasks in graphs and Pan et al. [23] recently introduced Adversarially Regularized Graph Autoencoder (ARGA) using GAN.

## 3. Problem Definition

Formally, a network with *N* nodes can be defined as G=(V,E), where $V=\{v_{i},\ldots ,v_{N}\}$ denotes the set of nodes and $E=\{e_{ij}\}$ is the set of edges. Incidentally, each node may be described by some features $X=\{x_{1},\ldots ,x_{N}\}$ where $x_{i}\in R^{D}$ defines a vector of real-values associated with node $v_{i}$ with *D*-dimension. Vectorizing the notations, $A=\left\{a_{1},\ldots ,a_{N}\right\}\in R^{N\times N}$ is the adjacency matrix of G. In this work, we consider the undirected and unweighted network G, such that $A_{ij}=1$ if $e_{ij}\in E$ otherwise 0. Given the network G, we aim to partition the nodes in G into *K* disjoint groups $\left\{c_{1},c_{2},\ldots ,c_{K}\right\}$ such that nodes grouped within the same communities share a similar connectivity pattern. We define the connectivity pattern by the node’s edge probability *p*. Specifically, $p_{in}$ is the probability of connecting between nodes of the same community and $p_{out}$ is the probability of connecting nodes between other communities. Consequently, the community structure is defined as,

(1) $$p_{in}>p_{out}.$$

We refer to this as the *modern view* community structure definition as suggested in [62]. This notion of community structure is a generalization to a probabilistic perspective.

Additionally, we further constrain our problem definition to the view of a generative model. Given a generative model, p(θ∣X,A) infers the model parameters θ from the observed network G. Concretely, we are interested to maximize,

(2) $$\arg\max_{\theta }p\left(A|\theta \right).$$

Under this optimization condition, a similar graph, $G^{\prime }$ with adjacency matrix $A^{\prime }$ can be generated from the same set of parameters such that $p(A^{\prime }|\theta )$ defines the reconstruction probability of the original adjacency matrix A. According to Bayesian principles, one can say that the model is a good model when $A^{\prime }≊A$ and satisfies the condition of having community structures as defined in Equation (1).

## 4. Model Description

### 4.1. Variational Graph Autoencoder for Community Detection

Kipf and Welling [21] introduced Variational Graph Autoencoder (VGAE) by replacing the encoder of Graph Autoencoder (GAE) [21,50] with a Graph Convolutional Network [14] and an inner product decoder. Formally, VGAE’s decoder can be defined as

(3) $$p\left(A|Z\right)=\prod_{i=1}^{N}\prod_{j=1}^{N}p\left(A_{ij}=1|z_{i}z_{j}\right)=\tau \left(z_{i}^{⊤}z_{j}\right),$$

where $Z\in R^{N\times F}$ is the latent representation with *N* nodes and *F* is the size of the latent representation, given as a hyperparameter. Additionally, we denote the latent representation of node $v_{i}$ as $z_{i}$ such that $\{z_{i},\ldots z_{N}\}\in Z$. The decoding process then follows a sampling process from the variational distribution q(·). Specifically, the model samples from a Gaussian prior distribution, $N(\cdot |\mu_{i},\sigma_{i}^{2}I)$ with mean μ, variance $\sigma^{2}$ and the identity matrix I. Samples are then mapped through a non-linear function denoted by τ(·). Most commonly, the non-linear function can be a logistic sigmoid function, $\tau \left(t\right)=1/\left(1+e^{-t}\right))$ or a ReLU function, ReLU(t)=max(0,t). The encoder is then defined as

(4) $$\begin{array}{c} q\left(Z|X,A\right)=\prod_{i=1}^{N}q\left(z_{i}|X,A\right)\\ q\left(z_{i}|X,A\right)=N\left(z_{i}|\mu_{i},\sigma_{i}^{2}I\right). \end{array}$$

The encoder q(·) is a variational distribution that approximates the true distribution p(·) [20,29]. By mathematical convenience, q(·) is usually a member of the exponential family. The mean μ and standard deviation σ are obtained through *amortization* using a two-layer GCN defined as,

(5) $$\mathrm{GCN}\left(X,A\right)=\hat{A}\tau \left(\hat{A}XW_{0}\right)W_{1},$$

where $\hat{A}$ is obtained through a *renormalization trick* [14], $\hat{A}=D^{-\frac{1}{2}}AD^{-\frac{1}{2}}$ and $\left\{W_{0},W_{1}\right\}$ are the trainable weight filters for each GCN layer. To train VGAE, we optimize the evidence lower bound (ELBO) L(·),

(6) $$\begin{array}{cc} \log p_{\theta }\left(X\right)\geq L\left(\theta ,\phi ;X\right) & =E_{q_{\phi }\left(Z|X,A\right)}\left[\log p_{\theta }\left(A|Z\right)\right]\\ \; & \;-D_{KL}[q_{\phi }\left(Z|X\right)||p_{\theta }\left(Z\right)]. \end{array}$$

$D_{KL}[q_{\phi }\left(\cdot \right)||p_{\theta }\left(\cdot \right)]$ defines the Kullback–Leibler (KL) divergence between $q_{\phi }\left(\cdot \right)$ and $p_{\theta }\left(\cdot \right)$. The lower bound can be maximized with respect to the variational parameters $\left(\theta ,\phi \right)=W_{i}$ via stochastic gradient descent. Here, the prior is defined as $p_{\theta }\left(Z\right)=\prod_{i=1}^{N}N\left(z_{i}|0,I\right)$, the isotropic Gaussian. Since this requires sampling from a Gaussian white noise, backpropagation from a stochastic variable is not trivial. Equation (6) via stochastic gradient descent. However, by applying a *reparameterization trick* [20], gradients can backpropagate to deterministic variables and stochastic variables can be effectively ignored.

Following prior work, Variational Graph Autoencoder for Community Detection (VGAECD) [22] generalizes the generation process of VGAE by introducing a mixture of Gaussians in the generation process (decoder). The generation process can be generalized to a mixture of Gaussians by introducing a community assignment parameter *c*. Specifically, we would like to calculate the joint probability distribution of p(a,z,c) such that

(7) $$\begin{array}{c} p(a,z,c)=p(a|z)p(z|c)p(c)\\ p(c)=Cat(\cdot |\gamma )\\ p\left(z|c\right)=N\left(\cdot |\mu_{c},\sigma_{c}^{2}I\right)\\ p\left(a|z\right)=\phi (z_{i}^{T}z;N\left(\cdot |\mu_{a},\sigma_{a}^{2}I\right)). \end{array}$$

For brevity, we drop the explicit subscript notation $z=z_{i}$ and $a=a_{i}$. In Equation (7), we obtain p(c) from the categorical distribution parameterized by γ with *K* communities. The parameter γ encodes our prior belief and is commonly initialized with a non-informative priors such as a uniform probability distribution. The reconstruction probability, p(a∣z) is the inner product between latent representations z parameterized by embeddings sampled from the Gaussian distribution. Effectively, two nodes $\nu_{i}$ and $\nu_{j}$ are more likely to have an edge $e_{ij}$ when their latent representations are closer to one another.

### 4.2. Linearization of the Encoder

VGAECD uses Graph Convolution layer (GCN) for its encoder to approximate parameters μ and σ. Albeit powerful, GCN is more computationally expensive due to its non-linearity and increase in training parameters required. Wu et al. [30] recently proposed a simplification of GCN by removing the non-linear component, τ(·), effectively linearizing GCN.

(8) $$\mathrm{SGC}\left(X,A\right)=\hat{A}\ldots \hat{A}\hat{A}XW^{\left(1\right)}W^{\left(2\right)}\ldots W^{\left(L\right)}.$$

Equation (8) describes SGC layer formally. In Equation (8), the non-linear function τ is removed and features from *L*-hop neighbors are accumulated. Equation (8) further simplifies to

(9) $$\mathrm{SGC}\left(X,A\right)={\hat{A}}^{L}XW,$$

with $W=W^{\left(1\right)}W^{\left(2\right)}\ldots W^{\left(L\right)}$. Similar to the *renormalization trick*, A two-layer L=2, SGC, ${\hat{A}}^{L}$ can be pre-computed before training. Extending Wu et al. [30]’s work, Salha et al. [31] demonstrated performance improvement upon linearization of the encoder on GAE and VGAE in link prediction and clustering tasks. From the perspective of graph signal processing, NT and Maehara [63] considered GCN & SGC to be equally powerful since both encoders resemble low-pass filters. Under the aforementioned motivation, we experimentally show that the linearization of the encoder reduces training time for convergence and time complexity. We further discuss about the implications of changing the encoder in Section 6.

### 4.3. Dual Optimization

In Section 4.1, we briefly discuss about the weakness of VGAECD. Formally, the objective function of VGAECD can be formulated into two losses,

(10) $$L=L_{\mathrm{recon}}+L_{\mathrm{comm}},$$

such that the reconstruction loss $L_{\mathrm{recon}}$ and the community’s quality loss, $L_{\mathrm{comm}}$ is minimized. It follows that optimizing the loss is not trivial. Given enough capacity, an autoencoder trained with stochastic gradient descent would favor optimizing its reconstruction loss ($L_{\mathrm{recon}}$), eventually leading to a posterior collapse [64]. Furthermore, as studied in [25,26,27], unsupervised deep learning algorithms tend to deviate from their main objective. They converge slowly especially when no guidance is given. In a similar fashion, we depict this problem exhibited by VGAECD in Figure 1. To rectify this issue, we propose a dual optimization algorithm based on Neural Expectation-Maximization (NEM) [28].

Unlike the Expectation-Maximization algorithm [65], Neural Expectation-Maximization (NEM) can be trained with gradient descent. As a result, VGAECD can be trained end-to-end. From Equation (7), the objective function can be defined as,

(11) $$\begin{array}{cc} \log p(a) & \geq L_{\mathrm{ELBO}}\left(a\right)=E_{q(z,c|a)}\left[\log\frac{p(a,z,c)}{q(z,c|a)}\right]. \end{array}$$

Reformulating Equation (11), we obtain

(12) $$L_{\mathrm{ELBO}}\left(a\right)=\underset{\mathrm{reconstruction}\;\mathrm{loss}}{\underset{︸}{E_{q(z,c|a)}\left[\log p\left(a|z\right)\right]}}-\underset{\mathrm{community}\;\mathrm{loss}}{\underset{︸}{D_{KL}[q(z,c|a)||p(z,c)]}}.$$

In Equation (12), by using a dual optimization process, the reconstruction loss is first optimized, followed by the community loss. This process is then repeated until convergence. Similar to [21,22], the reconstruction loss is minimized using binary cross-entropy and optimized using Adam [66]. The community loss is then minimized using an Expectation-Maximization (EM) algorithm [65] which guarantees a local optimum. Given $\psi_{i,k}=f_{\phi }{\left(\mu_{k}\right)}_{i}$ parameterized by ϕ and $\theta =\mu_{1},\ldots ,\mu_{K}$, the loss of our variational distribution follows,

(13) $$L_{\mathrm{comm}}\left(\theta ,\theta^{\mathrm{old}}\right)=\sum_{c}p\left(c|a,\psi^{\mathrm{old}}\right)\log p\left(a,c|\psi \right).$$

To optimize Equation (13), we use NEM as the optimization algorithm. First, we compute the expectation, obtaining γ, the soft assignment of each node $v_{i}$,

(14) $$\gamma_{i,k}:=p\left(c_{i,k}=1|z_{i},\psi_{i}^{\mathrm{old}\;}\right).$$

Next, the maximization step follows,

(15) $$\theta^{\mathrm{new}}=\theta^{\mathrm{old}}+\eta \frac{\partial L}{\partial \theta }$$

where

(16) $$\frac{\partial L}{\partial \mu_{k}}\propto \sum_{i=1}^{N}\gamma_{i,k}\left(\psi_{i,k}-a_{i}\right)\frac{\partial \psi_{i,k}}{\partial \mu_{k}}.$$

In Equation (15), η is the learning rate hyperparameter,. This process can be repeated *R*-times or until convergence. In practice, we found that R≈5 and η=0.01 would suffice to achieve convergence. The complete algorithm is described in Algorithm 1. For our decoding function $f_{\phi }\left(\cdot \right)$, we use a single layer Multilayer Perceptron (MLP) in this work.
**Algorithm 1** VGAECD-OPT     **Input:** Features X, Adjacency Matrix A, no. of comm. *K*, filter size D, number of epochs *L*,             NEM steps *R*.     **Output:** Community Assignment Probability γ and Reconstructed Adjacency matrix $\tilde{A}$1:π∼U(0,1)2:**for** l=1,…,L**do**3:    **for** i=1,…,N **do**4:        $\mu_{i}={\mathrm{SGC}}_{\mu }\left(x_{i},a_{i}\right)$5:        $\sigma_{i}={\mathrm{SGC}}_{\sigma }\left(x_{i},a_{i}\right)$6:        Sample $z_{i}∼N\left(\mu_{x}{|}_{i},\mathrm{diag}\left(\sigma_{x}^{2}{|}_{i}\right)\right)$7:        Obtain ${\tilde{a}}_{i}=\sigma \left(z_{i}^{⊤}z_{j}\right)$8:        Compute loss, $L_{\mathrm{ELBO}}$            ▹ From Equation (12)9:           and backpropagate gradients.10:    **end for**11:    **for** r=1,…,R **do**12:        Compute E-Step: γ, $\{\mu_{c}$, $\sigma_{c}\}$        ▹ From Equation (14)13:        Compute M-Step: $\mu_{c}$, $\sigma_{c}$, {γ}        ▹ From Equation (16)14:        Compute loss, $L_{\mathrm{comm}}$15:           and backpropagate gradients.16:    **end for**17:**end for**18:Extract community assignment ${arg max}_{k}\gamma$19:Return $\tilde{A}=\left\{{\tilde{a}}_{1},\ldots ,{\tilde{a}}_{N}\right\}$

**Figure 2:**
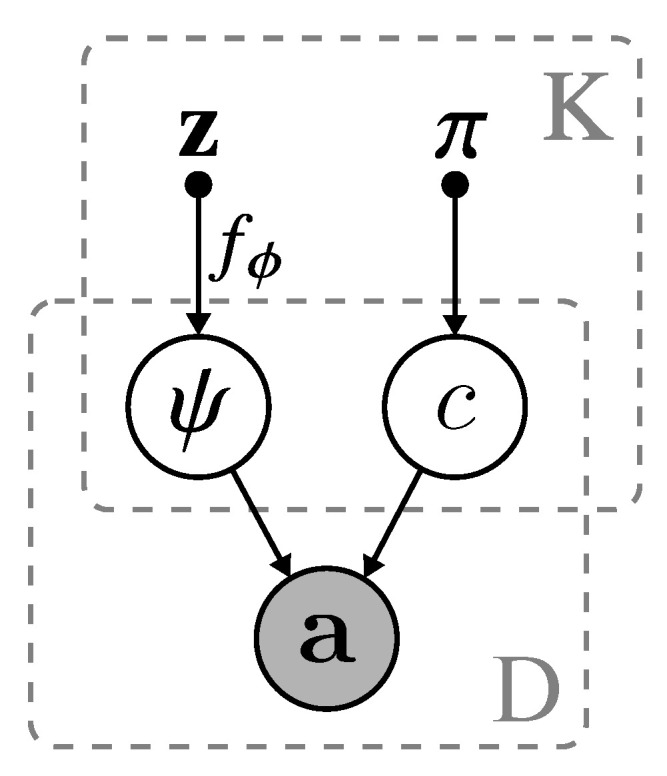
The probabilistic graphical model of VGAECD-OPT. The variable z is acquired from sampling of the variational distribution p(z∣c), π is the non-informative prior initialized uniformly. $f_{\phi }$ is the decoding function to obtain logits ψ. *K* is the number of clusters and *D* is the total number of data samples (i.e., |V|).

**Figure 3:**
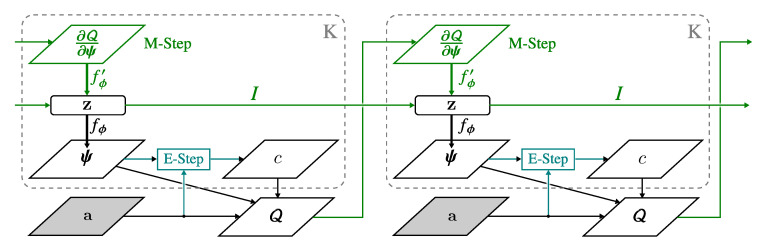
VGAECD optimized under Neural Expectation-Maximization algorithm (NEM). In the first iteration, the community assignment probability *c* is first computed (Expectation) followed by the Maximization step. We obtain probability ψ from the decoding function $f_{\phi }\left(\cdot \right)$ with embeddings z.

To explain this intuition, we refer to the theoretical formulation of VAE [66] and the bits-back argument [67]. Intuitively, while training, gradient signals would favor $L_{\mathrm{recon}}$ over minimizing $L_{\mathrm{comm}}$ when the model has high capacity. Consequently, each centroid $\mu_{k}$ is neglected; the centroid’s is randomly positioned in the embedding’s manifold and remain unoptimized. In an extreme case, the model would choose to collapse the posterior; resulting in a single cluster. To discourage this behavior, a dual optimization process allows gradient signals to be backpropagated to $\mu_{k}$ and $\sigma_{k}$. More specifically, turning $L_{\mathrm{comm}}$ to a variational EM optimization problem guarantees that the centroid’s embedding has a higher presence of encoding useful information. Moreover, this formulation retains the main characteristic of VAE without requiring auxiliary loss functions commonly found in other literature [24]. The complete algorithm can be found in Algorithm 1 with illustration shown in Figure 2 and Figure 3.

## 5. Experiments

In this section, we evaluate the optimized proposed algorithm (VGAECD-OPT). Similar to [22], we first evaluate on two benchmark graphs followed by real-world datasets. We note that all datasets have associated ground truths. In later subsections, we list our experiment settings and evaluation metric. We leave the discussion of our experimental findings to Section 6.

### 5.1. Synthetic Datasets

Two synthetic benchmarks are used in our evaluation. We consider the two most common benchmarks used for benchmarking community detection algorithms. Specifically, synthetic networks with community structures were generated with Girvan-Newman (GN) [7,35] benchmark and the LFR [56] benchmark. The result is a set of generated graphs with ground-truth labels (true partitions) used for evaluation purposes.

The GN benchmark is a variant of the planted *l*-partition model. Given a fixed number of communities c=4, and fixed number of nodes n=128, the GN benchmark graph generator generates a graph with *M* number of edges with an average degree k=16. A mixture variable $z_{\mathrm{out}}$ is manipulated from {1,…,8}, effectively controlling the connectivity pattern between intra-community $p_{in}$ and inter-community $p_{out}$ probabilities.

The LFR benchmark is an extension of the GN benchmark. It is considered to be more realistic than the GN benchmark while accounting for network heterogeneity and follows a power law distribution for the degree and community size distributions. The result is a generated network with variable communities of different sizes. To ensure consistency, default parameters were used [56]. These parameters are, number of nodes (N=1000), average degree (k=15), minimum ($c_{min}=30$) and maximum ($c_{max}=50$) number of nodes per community. The generation follows the *scale-free* parameters settings of exponents $\tau_{1}=-2$ and $\tau_{2}=-1$ respectively. On average, between 20 to 30 communities are generated.

### 5.2. Real-World Datasets

To evaluate performance of VGAECD-OPT, real-world datasets were used. These datasets are divided into two categories; networks with and without features. All datasets have ground-truth labels associated with them. The datasets are listed as follows:

- **Karate**: A social network that represents friendship among 34 members of a karate club at a US University observed by Zachary [1]. Community assignment corresponds to the clubs that members went to after the club split.
- **PolBlogs**: A network of political blogs assembled by Adamic and Glance [68]. The nodes are blogs, and web links between them are represented by its edge. These blogs have known political leanings and were labeled by hand by Adamic and Glance [68].
- **Cora**: A citation network with 2708 nodes and 5429 edges. Each node corresponds to a document and the edges are citation links [69,70]. Class labels correspond to each paper’s topic curated by Cora’s site portal [70] and were compiled by Sen et al. [69].
- **PubMed**: A network consisting of 19,717 scientific publications from PubMed database pertaining to diabetes classified into one of three classes (“Diabetes Mellitus, Experimental”, “Diabetes Mellitus Type 1”, “Diabetes Mellitus Type 2”). The citation network consists of 44,338 links. Each publication in the dataset is described by a TF-IDF weighted word vector from a dictionary, which consists of 500 unique words.

For starters, we perform experiments on datasets following Karrer and Newman [19]. These networks (Karate and PolBlogs) are featureless and only contain structural information. The Karate network is a commonly studied real-world network for community detection. Similar to [19], we consider the largest connected component and its undirected form for the PolBlogs dataset. Next, we used two networks containing features (Cora and PubMed) [10,14]. Table 1 summarizes the list of datasets and their respective properties.

### 5.3. Experimental Settings

For a baseline comparison, we chose to compare with VGAE and VGAECD. For generative models, we chose SBM [18], SBM (D.C) [19], VGAE and VGAECD as baseline comparisons. SBM and SBM (D.C) requires a specific optimization algorithm. In this case, it is optimized with Variational Expectation-Maximization (VEM) for the best performance. The encoder of VGAE and VGAECD consists of a 2-layer GCN (L=2) with configuration settings of (32-16), (32-16), (32-8), and (32-8) *D*-dimension for Karate, PolBlogs, Cora and PubMed respectively. Since VGAECD-OPT consists of only a single layer W, for a fair comparison, we use (16), (16), (8), and (8) for Karate, PolBlogs, Cora, and PubMed which are the deepest layer’s dimension in VGAECD. Additionally, we set the number of hops the same in Equation (9), i.e., L=2 and the fixed number of epochs to 200 [21].

For generative models, we chose SBM [18], SBM (D.C) [19], VGAE and VGAECD as baseline methods. SBM and SBM (D.C) employ different optimization strategies. For runtime feasibility, we have chosen to use a Markov Chain Monte Carlo (MCMC) sampling strategy. The encoder of VGAE and VGAECD consists of a 2-layer GCN with configuration settings of (32-16), (32-16), (32-8), and (32-8) *D*-dimension for Karate, PolBlogs, Cora and PubMed respectively. Since VGAECD-OPT consists of only a single layer W, for a fair comparison, we use (16), (16), (8), and (8) for Karate, PolBlogs, Cora, and PubMed which are the deepest layer’s dimension in VGAECD.

All experiments are conducted on a Linux Machine with Intel i9-7900X CPU @ 3.30GHz, 64GB @ 2666 MHz DDR3 memory and Nvidia GeForce GTX 1080Ti (12GB GPU memory) ×2 GPU with PyTorch framework. By default, all compatible algorithms were performed on GPU; otherwise, they are experimented using CPU computation.

### 5.4. Evaluation Metric

For evaluation purposes, we chose standard baseline metrics from [71]. These metrics are divided into two types. Specifically, the first three metrics have known ground truth, and the last three do not. In this case, the previous three metrics are useful to determine the quality of communities recovered.

- Accuracy measures the number of correctly classified clusters given the ground-truth. Formally, given two sets of community labels, i.e., *C* is the ground-truth and $C^{\prime }$ is the detected community labels, the accuracy can be calculated by,
  $$ACC\left(C^{\prime }\right)=\frac{\sum_{i=1}^{\left|C\right|}\delta \left(c_{i},c_{i}^{\prime }\right)}{\left|C\right|}\times 100\%.$$
  $c_{i}\in C,c_{i}^{\prime }\in C^{\prime }$, where δ(·) denotes the Kronecker delta, $\delta (c_{i},c_{i}^{\prime })=1$ when both labels matches and |·| denotes the cardinality of a set. For clustering tasks, accuracy is usually not emphasized as labels are known to oscillate between clusters.
- NMI and VI are based on information theory. NMI measures the ‘similarity’ between two community covers, while VI measures their ‘dissimilarity’ in terms of uncertainty. Correspondingly, a higher NMI indicates a better match between both covers while VI indicates the opposite. Formally [72],
  $$\mathrm{NMI}\left(C,C^{\prime }\right)=\frac{2I(C,C^{\prime })}{\left(H\left(C\right)+H\left(C^{\prime }\right)\right)}$$
  and
  $$\mathrm{VI}\left(C,C^{\prime }\right)=H\left(C\right)+H\left(C^{\prime }\right)-2I\left(C,C^{\prime }\right),$$
  where H(·) is the entropy function, and I$\left(C,C^{\prime }\right)=H\left(C\right)+H\left(C^{\prime }\right)-H\left(C,C^{\prime }\right)$ is the mutual information function.
- Modularity (Q) [73] measures the quality of a particular community structure when compared to a null (random) model. Intuitively, intra-community links are expected to be stronger than inter-community links. Specifically,
  $$Q=\frac{1}{2m}\sum_{ij}\left(A_{ij}-\frac{k_{i}k_{j}}{2m}\right)\delta \left(c_{i},c_{j}\right),$$
  where $A_{ij}-k_{i}k_{j}/2m$ measures the actual edge connectivity versus the expectation at random and $\delta (c_{i},c_{j})$ defines the Kronecker delta, where $\delta (c_{i},c_{j})=1$ when both node *i* and *j* belongs to the same community, and 0 otherwise. The modularity score Q∈[−1,1] approaches 1 when partitioning is close to optimum.
- Conductance (CON) [37,71] measures the separability of a community across the fraction of outgoing local volume of links in the community, which is defined as,
  $$\mathrm{CON}\left(C\right)=\frac{\sum_{i\in C,j\in C^{\prime }}A_{ij}}{\min(a\left(C\right),a\left(C^{\prime }\right))},$$
  where the nominator defines the total number of edges within community *C* and $a\left(C\right)=\sum_{i\in C}\left(j\in V\right)$ defines the volume of set C⊆V. A better local separability of community is achieved when the overall conductance value is the smallest.
- Triangle Participation Ratio (TPR) [71] measures the fraction of triads within the community *C*.
  $$\begin{array}{cc} \mathrm{TPR}(C) & =|\{v_{i}\in C,\{\left(v_{j},v_{k}\right):v_{j},v_{k}\in C,\\ \; & \left(v_{i},v_{j}\right),\left(v_{j},v_{k}\right),\left(v_{i},v_{k}\right)\in E\}\neq \emptyset \}|/|C|, \end{array}$$
  where *E* denotes the total number of edges in the graph *G*. A larger TPR value indicates a denser community structure.

## 6. Results and Discussion

In this section, we compare our proposed model (VGAECD-OPT) with several baseline methods. Among the generative models, SBM is an unsupervised model that does not use representation learning. For methods such as VGAE, DeepWalk, and node2vec, the latent representation is first learned then a clustering algorithm such as *k*-means is used. The * symbol denotes methods that were confined to structural information only.

We begin by discussing the stability performance of VGAECD-OPT in contrast to VGAECD in Section 6.1. In Section 6.2 we discuss the performance on several synthetic datasets. Section 6.3 provides in-depth discussion on VGAECD-OPT’s performance and Section 6.4 compares the runtime and time complexity of VGAECD-OPT against baseline methods. Finally, we end this section with a discussion on the limitations of the VGAE framework in Section 6.5.

### 6.1. Stability Performance

**Figure 4:**
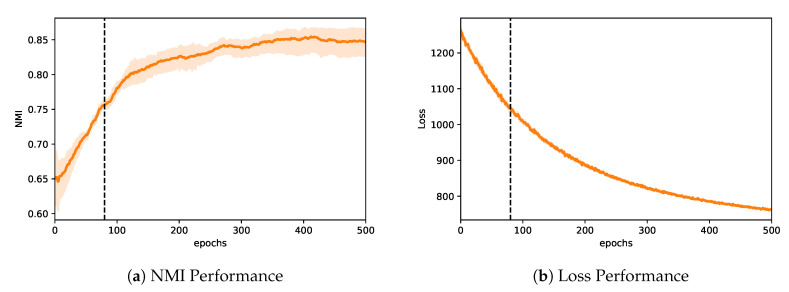
The proposed algorithm, VGAECD-OPT with Dual Optimization. In contrast to VGAECD, performance deviation is alleviated.

As discussed in Section 4.3 and illustrated in Figure 1, we show that optimizing VGAECD with standard SGD will eventually result in a deviation. To show that VGAECD-OPT does not exhibit such property, we illustrate VGAECD-OPT’s performance curve in Figure 4. Since VGAECD-OPT optimizes $L_{\mathrm{recon}}$ and $L_{\mathrm{comm}}$ loss in two different steps, VGAECD-OPT is more stable under the same training settings as VGAECD. On the contrary, our proposed algorithm achieves much higher NMI performance when initialized (epoch 0). At epoch 80, we achieve similar performance as VGAECD (Figure 1). After epoch 80, our algorithm continues to ascend without any decline in performance. Although we note that the rate of convergence for our loss is much slower in comparison to VGAECD, it does not hinder our main objective of achieving better community structure recovery.

### 6.2. Performance on Synthetic Datasets

**Figure 5:**
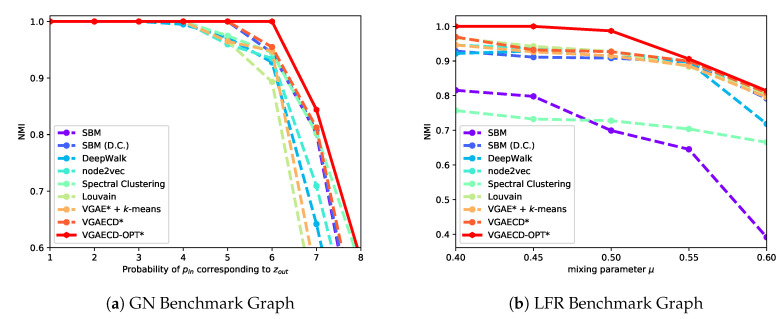
Comparative performance of VGAECD-OPT against VGAECD on Synthetic Networks.

For starters, VGAECD-OPT is evaluated against existing discriminative and generative methods on two synthetic datasets. For methods that were confined to structural information only (i.e., absence of features), we denote them with an asterisk (*) sign. The results performed on synthetic datasets are shown in Figure 5. As shown, VGAECD-OPT can recover community structures even in difficult settings (i.e., $z_{\mathrm{out}}\geq 6$ and μ≥0.50) meanwhile other algorithms exhibit difficulties. This proves that with linearization and dual optimization, VGAECD-OPT is much more stable than VGAECD. Its resiliency to posterior collapse has been mitigated, subsequently increasing the probability of community structure recovery.

### 6.3. Performance on Real-World Datasets

We now demonstrate the effectiveness of linearization and dual optimization approach towards real-world datasets. Table 2 and Table 3 demonstrates the performance of VGAECD-OPT on datasets without the presence of features. The arrows (↑ and ↓) indicate the direction towards better performance. For example, NMI (↑) indicates that the higher the value, the better the performance. Values marked in bold denote best-performing results. Additionally, we also note that the performance measure on ACC is subject to label oscillation. For instance, in a binary community detection task, communities are measured by an overlap between two covers (ground-truth and detected communities), but in classification tasks, exact label assignment assignments are accounted for (i.e., labeling a cat as a dog is a false positive).

As for competing baselines, we show that discriminative models such as Louvain’s algorithm, DeepWalk, and node2vec have competitive baseline performance. In general, Louvain’s method performs very well with modularity score. This is because Louvain’s method is by design an algorithm that maximizes modularity. However, this does not translate to true performance, as shown metrics with ground-truth measures (i.e., NMI, VI, and ACC). Node2vec and DeepWalk remain competitive in all datasets but performs poorly in Cora and PubMed datasets. These datasets contain features that determine the outcome of the algorithms’ performance. Among discriminative methods, Spectral Clustering has the highest variance in terms of performance. It performs extremely poor in PolBlogs like its generative model counterpart, the SBM. We reason that the algorithm is affected by hubs with high degrees like PolBlogs dataset [19]. As a result, both algorithms pick these hubs as single node communities resulting in poorer performance.

On the other hand, generative models such as SBM performed poorly, mainly when datasets such as Cora and PubMed are used. Since SBM does not support features, it is difficult for SBM to thrive, especially when these datasets are feature-driven [63]. Indeed, with VGAE-based approaches, the performance increases significantly. Most importantly, we note that VGAECD-OPT achieves the best performance among other variants.

In Karate dataset, it can be observed that VGAECD-SGC* and VGAECD-OPT* both performed poorer than VGAECD* in terms of NMI, VI, and ACC. Further analysis shows that the non-linearity of VGAECD was a contributing factor to its higher performance. However, as we demonstrate in Table 3, Table 4 and Table 5, the presence of non-linearity was mostly negligible. Instead, the NMI performance improved when VGAECD adopts an SGC encoder. For baseline purposes, we introduced VGAECD-SGC, which comes with a linearized encoder but an absent dual optimizer. Coupled with dual optimization, VGAECD-OPT consistently shows improvements in contrast to VGAECD. We describe this with Equation (13) such that $\theta >\theta^{\mathrm{old}}$ ensures each new community membership proposal follows proportionally to the loss.

### 6.4. Time Complexity Analysis

We now discuss the time complexity and runtime of the proposed algorithm. We divide our runtime analysis into four parts. In the first part, we will discuss the convergence rate of our proposed method. The second part analyzes the runtime performance of all methods on real-world datasets. The third part explores the scalability performance of our proposed method on synthetic networks. Finally, we present our analysis of the time complexity of all methods.

To measure convergence rate, we introduced an early stopping criterion (The early stopping criterion serves the purpose of measuring convergence rate only. In practice, algorithms run with a fixed number of epochs); when a specific NMI threshold is achieved, we terminate the algorithm. This allows fairer comparison since VGAECD-SGC and VGAECD-OPT converges faster than VGAECD. We present this result in Table 6. We show a marginal improvement in speed when the encoder has been replaced with a linear encoder. Coupled with a dual optimization process, we can obtain a faster convergence rate, resulting in a fewer number of training iterations. For Karate dataset, VGAECD-OPT is ahead by 1 s, whereas in the Cora dataset, it achieved a speedup of almost 2×. In contrast to VGAECD-SGC, the proposed algorithm is faster on Karate and Cora datasets but is slower on PolBlogs on average run time. We find this to be insignificant since the standard deviation is more unstable.

For complete runtimes analysis, we allow all VGAE variants to complete 200 epochs without early stopping. We present these results in Table 7. Overall, the fastest algorithm is Louvain’s method, which has been shown to run near-linear time in a very sparse network [7]. In the worst case, it performs with time complexity of O(NlogN) as presented in Table 8. On the other hand, our proposed algorithm performs better than state-of-the-art representation learning methods such as DeepWalk and node2vec on all real-world datasets (Karate, PolBlogs, Cora, and PubMed) despite being a generative model.

**Figure 6:**
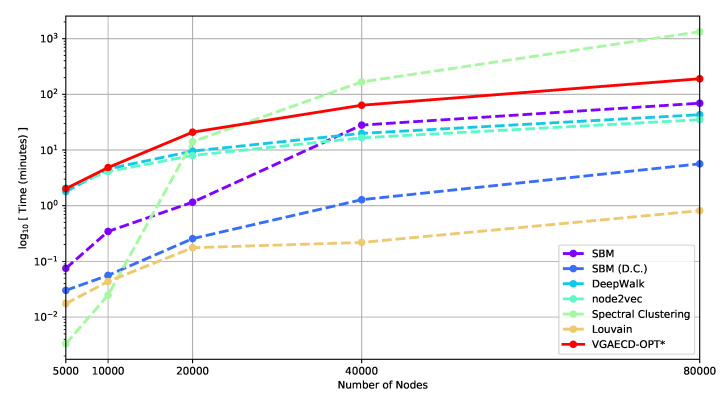
Runtime of VGAECD-OPT & baseline methods on LFR benchmark graphs.

To demonstrate runtime scalability, Figure 6 shows the algorithm’s expected runtime as the number of nodes and community increases. Each network is generated from an LFR benchmark with standard parameters (see Section 5.3), but with a variable number of nodes and communities. The resulting network is summarized in Table 9. Due to the nature of our proposed method being a generative model, the runtime performance approximately polynomial in runtime. Although SBM is a generative model, it employs different optimization strategies. For instance, the original implementation by Karrer and Newman [19] struggles beyond 5000 number of nodes. To overcome this, we used a Markov Chain Monte Carlo sampling strategy to obtain reasonable runtime results. When nodes and communities are fewer than 10,000 and 10, respectively, the performance of our method is comparable to DeepWalk and node2vec. This is because both discriminative methods do not account for communities and *k*-means is used instead, resulting in faster runtimes.

We now analyze the time complexity of VGAECD-OPT. From Algorithm 1, the encoder has a time complexity of $O(2N^{2}XD^{l})$ where *N* is the number of nodes, *D* is the size of the trainable graph filter, *l* is the number of linear layers, and *X* is the dimension of each node features. Since the number of filters is constant with respect to the number of layers, we have l=1. The constant 2 accounts for the computation of μ and σ. If we assume that the adjacency matrix is sparse, we can have an encoder with a complexity of O(NXD). With NEM, we introduce two additional steps, which has a time complexity of O(2NK) for performing the Expectation-Maximization steps. Given many samples, we can further simplify this to O(NK). With an inner product decoder, it has a time complexity of $O(N^{2})$. Overall, the final time complexity for one epoch is $O\left(NXD\right)+O\left(NK\right)+O\left(N^{2}\right)$. In comparison, VGAECD has a time complexity of $O\left(NXD^{2}\right)+O\left(N^{2}\right)$ due to its two-layer GCN architecture. Thus, we can conclude that VGAECD-OPT is relatively competitive with VGAECD in terms of time complexity.

We list a summary of the competing method’s time complexity in Table 8. Additionally, we note the following notations: *N*—number of nodes, γ—the number of random walks, *T*—walk length, *W*—window size, *D*—the representation size and *K*—number of communities.

### 6.5. Limitations of VGAE Framework

Overall, VGAE and its variants have proven to be an effective algorithm for learning networks with features. In this section, we highlight one shortcoming of VGAE that remains a challenge. In particular, VGAE has scaling difficulties. In VGAE, the inner product decoder uses a cross-entropy loss function. Unfortunately, this requires a dense by dense matrix multiplication, which requires a significant amount of memory for backpropagation purposes. In other literature, methods such as LINE [50] and DeepWalk [12] employs a negative sampling loss function for link prediction. In VGAE’s case, such implementation is not trivial as the task differs (reconstruction vs. link prediction). To explain this, let us consider an undirected unweighted graph G. One can observe that negative sampling considers the connectivity of each node by considering edges that are present and absent, $e_{ij}\in \left\{0,1\right\}$. Formally, negative sampling can be defined as

(17) $$\log\tau \left(z_{j}^{⊤}\cdot z_{i}\right)+\sum_{i=1}^{K}E_{v_{n}∼p_{n}\left(v\right)}\left[\log\tau \left(-z_{n}^{⊤}\cdot z_{i}\right)\right].$$

Here, we consider positive samples (edges) in the first term of Equation (17) with $z_{i}$ being the representation of node $v_{i}$ and negative samples in the second term. τ(·) denotes the non-linear function and *K* defines the number of negative samples which are drawn from some probability distribution $p_{n}\left(v\right)$. The first term of Equation (17) defines the likelihood of positive samples inducing an edge while the second term negates such probability. In other words, the loss function in Equation (17) induces separation of the positive samples from the negative samples, in such a way that the representations of each positive edge would stay further apart from negative edges. In DeepWalk and LINE, the effectiveness of negative sampling is highly dependent on the context (random walker’s chain). Under the current VGAE framework, such a context does not exist. Implementing this in VGAE is non-trivial. Hence, to scale our model, we need more efficient decoders, which remains a challenging task.

## 7. Conclusions

In this paper, we demonstrate that VAE and its variants (including VGAE & VGAECD) have a high tendency to favor minimization of the reconstruction loss over a clustering loss. As a result, it performs poorer as training prolongs overtime. To rectify this problem, we propose a dual optimization approach for optimizing VGAECD. We experimentally show the effectiveness of our dual optimization approach on VGAECD, allowing us to outperform its previous achievements. Moreover, to increase the speed of learning, we follow new practices of linearizing the encoder. Although the performance gain is marginal in terms of community detection, it has reduced the number of learnable parameters, which results in faster convergence and training speed.

## Figures and Tables

**Table 1:** Empirical network datasets.

Dataset	Type	Nodes	Edges	Communities (*K*)	Features
Karate	Social	34	78	2	*N/A*
PolBlogs	Blogs	1222	16,717	2	*N/A*
Cora	Citation	2708	5429	7	1433
PubMed	Citation	19,717	44,338	3	500

**Table 2:** Experimental results on karate dataset.

	NMI (↑)	VI (↓)	ACC (↑)	Q (↑)	CON (↓)	TPR (↑)
Spectral Clustering	0.7323	0.8742	0.6765	0.3599	0.1313	0.9403
Louvain	0.4900	1.5205	0.3235	**0.4188**	0.2879	0.7333
DeepWalk	0.7198	0.8812	0.9353	0.3582	0.1337	0.9353
node2vec	0.8372	0.8050	0.9706	0.1639	0.4239	0.4549
Stochastic Blockmodel	0.0105	1.1032	0.4412	−0.2084	0.7154	0.4034
Stochastic Blockmodel (D.C)	0.8372	0.8050	0.9706	0.3718	**0.1282**	**0.9412**
VGAE* + *k*-means	0.6486	0.8189	0.9647	0.3669	0.1295	0.9407
VGAECD*	**1.0000**	**0.6931**	**1.0000**	0.3582	0.1412	**0.9412**
VGAECD-SGC*	0.8372	0.8050	0.9706	0.3714	**0.1282**	0.9409
VGAECD-OPT*	0.8372	0.8050	0.9706	0.3742	**0.1282**	0.9409

**Table 3:** Experimental results on PolBlogs dataset.

	NMI (↑)	VI (↓)	ACC (↑)	Q (↑)	CON (↓)	TPR (↑)
Spectral Clustering	0.0014	1.1152	0.4828	−0.0578	0.5585	0.7221
Louvain	0.6446	1.0839	0.9149	0.2987	0.8130	0.1922
DeepWalk	0.7367	1.0839	0.9543	0.0980	0.3873	0.6870
node2vec	0.7545	0.8613	0.9586	0.1011	0.3827	0.6863
Stochastic Blockmodel	0.0002	1.2957	0.4905	−0.0235	0.5329	0.5657
Stochastic Blockmodel (D.C)	0.7145	0.8890	0.9496	**0.4256**	**0.0730**	0.8101
VGAE* + *k*-means	0.7361	0.8750	0.9552	0.4238	0.0752	0.8089
VGAECD*	0.7583	0.8583	**0.9601**	0.4112	0.0880	0.7913
VGAECD-SGC*	0.7235	0.8808	0.9492	0.4248	0.0735	**0.8142**
VGAECD-OPT*	**0.7620**	**0.8558**	**0.9601**	0.4252	0.0734	0.8086

**Table 4:** Experimental results on Cora dataset.

	NMI (↑)	VI (↓)	ACC (↑)	Q (↑)	CON (↓)	TPR (↑)
Spectral Clustering	0.2623	**2.4183**	0.1770	0.0011	0.8527	0.0577
Louvain	0.4336	4.0978	0.0081	**0.8142**	0.0326	0.2821
DeepWalk	0.3796	2.7300	0.1626	0.6595	**0.0396**	0.4949
node2vec	0.3533	2.9947	0.1359	0.6813	0.1078	0.4902
Stochastic Blockmodel	0.0917	3.5108	0.1639	0.4068	0.4280	0.3376
Stochastic Blockmodel (D.C.)	0.1679	3.4547	0.1176	0.6809	0.1736	0.5112
VGAE* + *k*-means	0.2384	3.3151	0.1033	0.6911	0.1615	0.4906
VGAE + *k*-means	0.3173	3.1277	0.1589	0.6981	0.1517	0.5031
VGAECD*	0.2822	3.1606	0.1532	0.6674	0.1808	0.5076
VGAECD	0.5072	2.7787	0.1101	0.7029	0.1371	0.4987
VGAECD-SGC*	0.3003	3.1734	0.1418	0.6116	0.2125	0.4479
VGAECD-SGC	0.5170	2.7707	0.2610	0.7138	0.1345	0.5053
VGAECD-OPT*	0.3735	2.4200	0.2717	0.4930	0.1792	0.4921
VGAECD-OPT	**0.5437**	2.6877	**0.3190**	0.7213	0.1227	**0.5324**

**Table 5:** Experimental results on PubMed dataset.

	NMI (↑)	VI (↓)	ACC (↑)	Q (↑)	CON (↓)	TPR (↑)
Spectral Clustering	0.1829	**1.4802**	0.3405	0.4327	**0.0249**	0.1850
Louvain	0.1983	3.6667	0.0954	**0.7726**	0.1388	0.1592
DeepWalk	0.2946	1.7865	0.3101	0.5766	0.0499	0.2461
node2vec	0.1197	1.9849	0.2228	0.3501	0.3170	0.2269
Stochastic Blockmodel	0.0004	1.9340	0.3080	−0.1620	0.1038	0.1965
Stochastic Blockmodel (D.C.)	0.1325	2.0035	0.3118	0.5622	0.8121	0.2441
VGAE* + *k*-means	0.2041	1.8096	0.3724	0.5273	0.1320	0.2898
VGAE + *k*-means	0.1981	1.8114	0.2751	0.5297	0.1283	0.2900
VGAECD*	0.1642	1.8320	0.1956	0.4966	0.1252	0.2692
VGAECD	0.3252	1.7056	**0.4216**	0.6878	0.1636	**0.4827**
VGAECD-SGC*	0.2350	1.8630	0.4155	0.5501	0.1163	0.2524
VGAECD-SGC	0.2948	1.7960	0.2396	0.5413	0.1044	0.2463
VGAECD-OPT*	0.2505	1.8517	0.3223	0.5853	0.0800	0.2519
VGAECD-OPT	**0.3552**	1.7082	0.3223	0.5378	0.0830	0.2446

**Table 6:** Convergence rate of VGAECD-OPT vs. VGAECD.

	Karate	PolBlogs	Cora	PubMed
VGAECD	3.3297 ± 0.0336	8.6538 ± 0.2808	6.6419 ± 0.1886	82.2131 ± 0.1321
VGAECD-SGC	2.8960 ± 0.0320	4.7735 ± 0.0372	3.9832 ± 0.0209	68.2313 ± 0.0332
VGAECD-OPT	2.1015 ± 0.0100	5.0768 ± 0.0120	3.6996 ± 0.0275	67.8840 ± 0.0313

**Table 7:** Runtime comparison between VGAECD-OPT and baseline methods in (s)econds.

	Karate	PolBlogs	Cora	Pubmed
Spectral Clustering	0.0111 ± 0.0004	0.0981 ± 0.0129	0.1932 ± 0.0247	14.835 ± 0.1107
Louvain	0.0020 ± 0.0003	0.2765 ± 0.0204	0.2571 ± 0.0201	3.1068 ± 0.0021
DeepWalk	0.2805 ± 0.0204	29.3969 ± 1.7295	60.2633 ± 3.3005	446.1594 ± 1.5393
node2vec	4.1691 ± 0.0071	73.8038 ± 0.2477	59.8279 ± 0.0681	451.6884 ± 0.1085
Stochastic Blockmodel	0.2126 ± 0.0030	0.2831 ± 0.0078	7.4576 ± 4.7685	6.3896 ± 3.9298
Stochastic Blockmodel (D.C.)	0.1452 ± 0.0336	0.2344 ± 0.0796	3.2463 ± 1.7783	3.3545 ± 2.7707
VGAE* + *k*-means	3.2319 ± 0.1204	18.6163 ± 0.3803	6.5510 ± 0.2043	93.4253 ± 0.2476
VGAECD*	3.3363 ± 0.0539	21.3191 ± 0.2571	7.4428 ± 0.1177	93.5190 ± 0.3785
VGAECD-SGC*	3.3503 ± 0.0418	19.0820 ± 0.0386	4.7377 ± 0.1175	89.8966 ± 0.0844
VGAECD-OPT*	2.4467 ± 0.0238	20.2052 ± 0.0649	7.4037 ± 0.0342	92.1212 ± 0.1192

**Table 8:** Time complexity.

Method	Complexity
Spectral Clustering	$O(N^{3})$
Louvain	O(NlogN)
DeepWalk	O(γNTW(D+DlogN))
node2vec	O(γNTW(D+DlogN))
Stochastic Blockmodel	$O(N^{2}K)$
Stochastic Blockmodel (D.C.)	$O(N^{2}K)$
VGAE + *k*-means	$O\left(NXD^{2}\right)+O\left(NK\right)$
VGAECD	$O\left(NXD^{2}\right)+O\left(N^{2}\right)$
VGAECD-SGC	$O\left(NXD\right)+O\left(N^{2}\right)$
VGAECD-OPT	$O\left(NXD\right)+O\left(NK\right)+O\left(N^{2}\right)$

**Table 9:** Networks used.

Number of Nodes	Edges	Communities
5000	74,278	5
10,000	148,427	10
20,000	295,857	20
40,000	599,396	40
80,000	1,189,991	80
